# An RNAi Screen for Genes Required for Growth of *Drosophila* Wing Tissue

**DOI:** 10.1534/g3.119.400581

**Published:** 2019-10-13

**Authors:** Michael D. Rotelli, Anna M. Bolling, Andrew W. Killion, Abraham J. Weinberg, Michael J. Dixon, Brian R. Calvi

**Affiliations:** * Department of Biology, Indiana University, Bloomington, IN 47405 and; † Melvin and Bren Simon Cancer Center, Indiana University, Indianapolis, IN 46202

**Keywords:** Drosophila, wing disc, tissue growth, polyploid, endoreplication

## Abstract

Cell division and tissue growth must be coordinated with development. Defects in these processes are the basis for a number of diseases, including developmental malformations and cancer. We have conducted an unbiased RNAi screen for genes that are required for growth in the *Drosophila* wing, using GAL4-inducible short hairpin RNA (shRNA) fly strains made by the Drosophila RNAi Screening Center. shRNA expression down the center of the larval wing disc using *dpp-GAL4*, and the central region of the adult wing was then scored for tissue growth and wing hair morphology. Out of 4,753 shRNA crosses that survived to adulthood, 18 had impaired wing growth. FlyBase and the new Alliance of Genome Resources knowledgebases were used to determine the known or predicted functions of these genes and the association of their human orthologs with disease. The function of eight of the genes identified has not been previously defined in *Drosophila*. The genes identified included those with known or predicted functions in cell cycle, chromosome segregation, morphogenesis, metabolism, steroid processing, transcription, and translation. All but one of the genes are similar to those in humans, and many are associated with disease. Knockdown of *lin-52*, a subunit of the Myb-MuvB transcription factor, or β*NACtes6*, a gene involved in protein folding and trafficking, resulted in a switch from cell proliferation to an endoreplication growth program through which wing tissue grew by an increase in cell size (hypertrophy). It is anticipated that further analysis of the genes that we have identified will reveal new mechanisms that regulate tissue growth during development.

Tissues must grow to a specific size and shape for proper development. This process is regulated by signals that coordinate cell division, cell growth, and cell death across tissues in both time and space (Vollmer *et al.* 2017). Perturbations in these tissue growth programs are known causes of developmental malformations and cancer (Hanahan and Weinberg 2011; Khetarpal *et al.* 2016; Parvy *et al.* 2018). While many tissues grow through an increase in cell number by mitotic cell proliferation, others grow by an increase in cell size through alternative polyploid endoreplication cycles (Øvrebø and Edgar 2018; Gjelsvik *et al.* 2019). Much remains unknown, however, about how tissue growth is regulated to achieve normal organ size and shape. To identify genes that participate in this process, we have conducted an RNAi screen in the *Drosophila* wing.

The *Drosophila* wing disc has been an important model for developmental regulation of tissue growth and patterning (Hariharan and Serras 2017; Vollmer *et al.* 2017). Wing discs originate as a group of ∼30-50 cells during embryogenesis, and then grow by cell proliferation during larval stages, ultimately reaching a size of ∼30,000-50,000 cells (Worley *et al.* 2013). During larval stages, the developmental axes of the wing disc and the fates of different cells are progressively patterned by developmental signaling pathways (Ruiz-Losada *et al.* 2018). During subsequent pupal stages, cell proliferation ceases and the wing disc tissue differentiates and everts to form different parts of the wing, wing hinge, and notum of the fly thorax (Aldaz and Escudero 2010). Early experiments using genetic and surgical manipulation of wing discs revealed fundamental principles of growth, patterning, and regeneration (Garcia-Bellido *et al.* 1973; Bryant 1975; Kiehle and Schubiger 1985; Maves and Schubiger 2003; Neto-Silva *et al.* 2009). Wing discs have continued to be important models for the discovery of conserved pathways that control tissue patterning and growth, including those that regulate the compensatory proliferation of cells in response to tissue damage (Neufeld *et al.* 1998; De La Cova *et al.* 2004; Hariharan and Serras 2017).

To identify genes that are important for tissue growth, we have screened a collection of GAL4-inducible short hairpin RNA (shRNA) strains for their effect on the *Drosophila* wing (Ni *et al.* 2011; Heigwer *et al.* 2018). We recently conducted a candidate shRNA screen of 240 genes, which RNA-Seq had shown are expressed at lower levels in endoreplicating cells in culture. This candidate screen showed that knockdown of genes in a CycA – Myb – Aurora B pathway induces cells in the wing and other tissues to switch to an alternative endoreplication growth program (Rotelli *et al.* 2019). Here, we report the results of a random screen of 5,260 additional shRNA strains, which has identified 18 genes whose knockdown impairs wing growth. The function of eight of the genes recovered in this screen has not been previously defined in *Drosophila*. The human orthologs of some of these genes are associated with disease, including those that manifest as tissue undergrowth or cancer. Immunofluorescent analysis of wing discs showed that knockdown of two genes induced a switch from mitotic cell divisions to polyploid endoreplication cycles, providing an inroad to understanding the regulation of these alternative growth programs.

## Materials and Methods

### Drosophila genetics

*Drosophila* were raised on BDSC standard cornmeal medium at 25°. The TRiP *UAS-shRNA Drosophila* strains were made by the Drosophila RNAi Screening Center (DRSC) (Ni *et al.* 2011), and were obtained from the Bloomington Stock Center (BDSC, Bloomington, IN) (Cook *et al.* 2010). The *P{GAL4-dpp.blk1]40C.6 / TM3 Sb Ser and P{GAL4-dpp.blk1]40C.6 UAS-mRFP / TM6 Tb* strains were constructed from the Bloomington stock *P{GAL4-dpp.blk1]40C.6 / TM6 Tb* (#1551). See Table S1 for a complete list of strains and stock numbers.

### Adult wing screen

**Figure 1:**
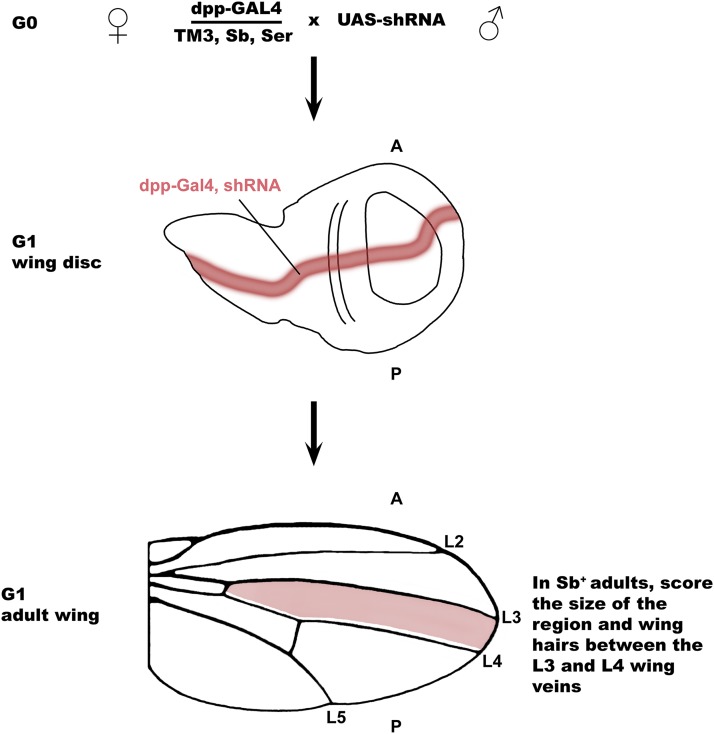
Screen strategy to identify genes required for wing growth. The dpp-Gal4 / TM3 Sb Ser strain females was crossed to different *UAS-shRNA* strain males from the TRiP collection. The *UAS-shRNA / +* ; *dpp-GAL4 / +* progeny have expression of the shRNA expression in a dpp-GAL4 expression domain along the anterior-posterior boundary of the larval wing disc (red), which in the wing pouch is fated to become the region of the adult wing between longitudinal wing veins 3 and 4 (L3 and L4) (red shading). The L3-L4 intervein region of these *UAS-shRNA / +* ; *dpp-GAL4 / +* progeny (Sb^+^ phenotype) was scored for total area and wing hair size, organization, and morphology relative to other wing regions, with *UAS-shRNA / +* ; *TM3 Sb Ser / +* (Sb^-^ phenotype) siblings serving as additional internal controls.

**Figure 2:**
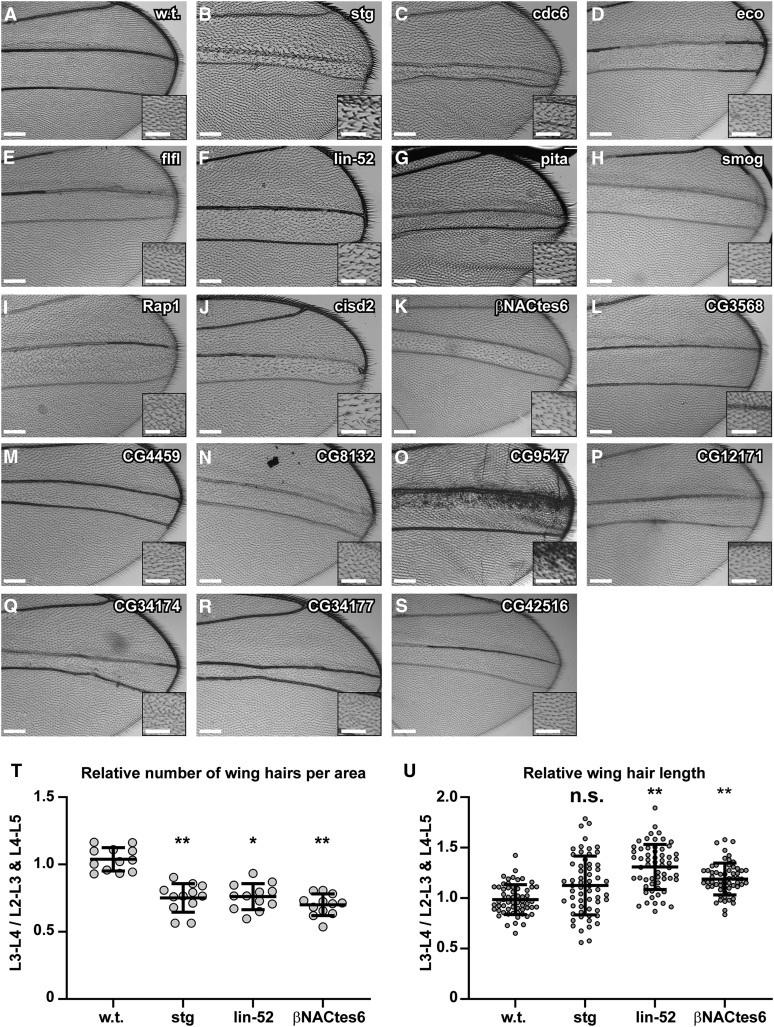
Adult wing phenotypes of shRNA strains that impaired growth. (A – S) Bright field images of adult wings from a wild type *dpp-GAL4 /+* control (A) or after expression of a *UAS-shRNA* targeting the indicated gene (B-S). Insets are higher magnifications to show wing hair phenotypes. Shown are the dorsal sides of the wings with anterior up. Scale bars are 150 μm for main panels and 75 μm for insets. (T, U) The length and number of wing hairs per unit area (hair density) were measured using the ImageJ plug in Fiji-wing. (T) Number of wing hairs per area from the L3-L4 intervein region divided by that in the L2-L3 + L4-L5 intervein regions of the same wings (N = three wings, with four L3-L4 areas and two L2-L3 + two L4-L5 areas per wing, **P ≤ 0.01, *P ≤ 0.05). (U) Length of wing hairs in the L3-L4 intervein region divided by that in the L2-L3 + L4-L5 intervein regions of the same wings (N = three wings with n = 20 hairs for L3-4 and 10 hairs for L2-L3 + 10 hairs for L4-L5 per wing. ** = *P* ≤ 0.01, * = *P* ≤ 0.05, n.s. = not significant, by Student’s *t*-test).

The *UAS-shRNA* strains were crossed to *P{GAL4-dpp.blk1]40C.6 / TM3 Sb Ser*, and the wings of adult *UAS-shRNA / +* ; *dpp-GAL4 / +* progeny were scored for reduced growth of the region between longitudinal wing veins 3 (L3) and 4 (L4), a region that is also known as the first posterior compartment (FPC) (Ferris 1950), although it emanates from the anterior lineage compartment of the wing disc (Figure 1). The *shRNA / +*; *TM3 Sb Ser* siblings from this cross served as internal negative controls. The *shRNA* strains found to affect wing growth / hair morphology in the primary screen were retested and scored for expressivity and penetrance. Adult wings were dry mounted with coverslips and imaged under bright field on a Leica DMRA2 microscope (Figure 2).

This L3-L4 intervein region was also scored for aberrant wing hair (trichome) morphology, spacing, and planar patterning relative to the other areas of the same wing. Relative number of wing hairs per area (hair density) was measured using ImageJ (v1.5e) (https://imagej.nih.gov/ij/) with the FijiWings plugin (v2.2) (Dobens and Dobens 2013). For three separate wings, the trichome density from four selected areas of the L3-L4 intervein region was compared to the average of two selections of the same size from the L2-L3 intervein region and two selections from the L4-L5 intervein region. The average length of twenty wing hairs from the L3-L4 intervein region were compared to that of ten wing hairs from the L2-L3 intervein region and ten wing hairs from the L4-L5 intervein region, for three separate wings. Relative wing hair density and relative length values were plotted using GraphPad Prism (version 7.04).

### Antibody labeling and immunofluorescent microscopy

**Figure 3:**
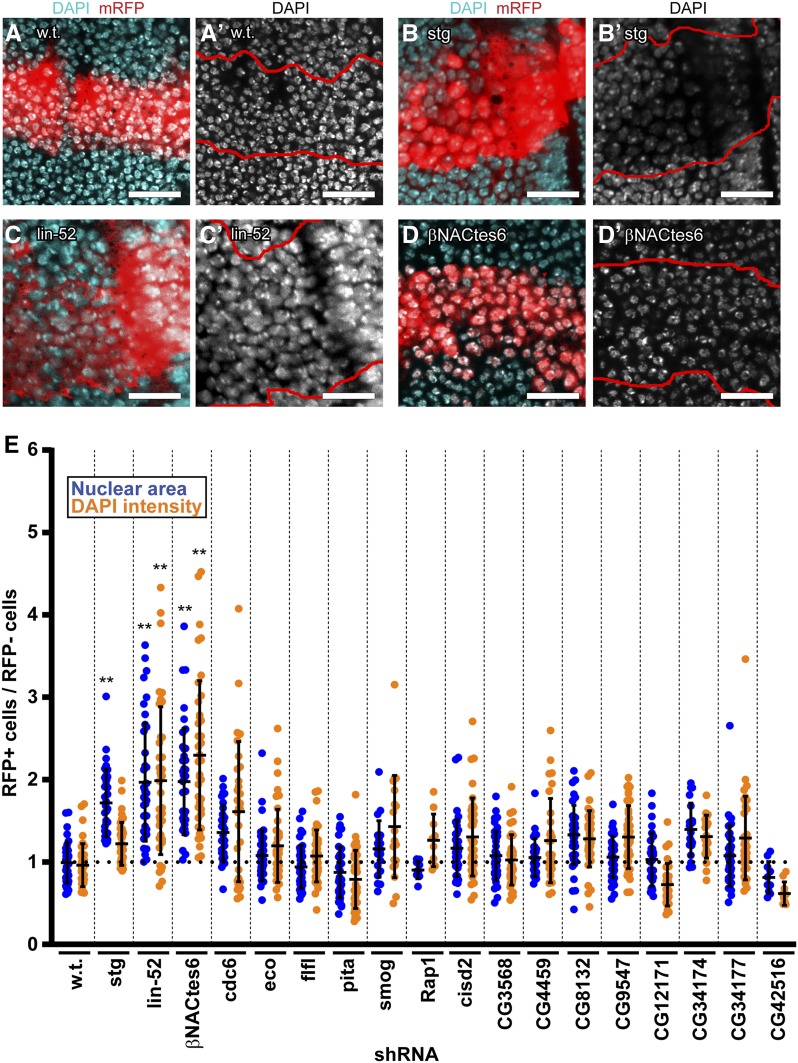
Immunofluorescent analysis of the effect of gene knockdown on ploidy of wing imaginal discs. (A-D’) Confocal images of wandering third instar wing discs labeled with antibodies against mRFP and the nuclear DNA dye DAPI, from *UAS-mRFP / +*; *dpp-GAL4 / +* controls (A,A’), or after knockdown of *stg* (B, B’), *lin-52* (C,C’) or β*Nactes6* (D,D’). The red *UAS-mRFP* reporter expression indicates those cells that express *dpp-GAL4*, which is demarcated by red outlines in A’, B’, C’, and D’, with DAPI labeled nuclei shown in black and white. (E) Quantification of the nuclear size and DAPI fluorescence of shRNA expressing cells (RFP+) were measured and normalized to cells outside of the *dpp-GAL4* domain in the wing pouches of same wing disc. *lin-52* and β*Nactes6* knockdown resulted in significantly increased nuclear size and DNA content, whereas *stg* knockdown had increased nuclear size but not DNA content (N = two discs, with a 20-40 RFP+ and 20-40 RFP- cells scored per disc, ** = *P* ≤ 0.01 by Student’s *t*-test).

For quantification of ploidy in Figure 3, wing imaginal discs from Tb+ 3^rd^ instar larvae were dissected and fixed as described (Schwed *et al.* 2002). Discs were labeled with rabbit anti-dsRed (Clontech, 632496) (1:400) and secondary anti-rabbit Alexa Fluor 568 (1:500) (Invitrogen), and stained with DAPI (0.5μg/ml). Discs were imaged on a Leica SP5 confocal and Leica DMRA2 widefield epifluorescence microscope. ImageJ was used to quantify nuclear area and total DAPI fluorescence. The nuclear area and DAPI intensity of cells within the RFP+ *dpp* expressing stripe were normalized to cells outside of the stripe in the same wing discs (RFP + cells / RFP- cells in Figure 3). The cells in the wing pouch area of each wing disc were scored, excluding the zone of non-proliferating cells, which are arrested in G1 and G2 phases of the cell cycle (Johnston and Edgar 1998).

### Statistical Analysis

For Figures 2T and 2U, statistical significance was determined by two-tailed Student’s *t*-tests using Microsoft Excel (version 15.0.4753.1000). For Figure 3E, the mean and standard deviation for nuclear size and DNA ploidy were measured for wild type and each shRNA knockdown. The significance of the difference between each shRNA knockdown and the wild type control was assessed by a two-tailed Student’s *t*-test with cut off of *P* ≤ 0.01.

### Data Availability

A list of fly strains screened can be found in Table S1. All fly strains are publicly available from the BDSC. All fly strains and reagents generated in this study will be made freely available upon request. Supplemental material available at FigShare: https://doi.org/10.25387/g3.8309594.

## Results and Discussion

### Overview of screen strategy and results

Our goal was to identify genes that are required for cell proliferation and growth. To do this, we expressed a collection of GAL4-inducible short hairpin RNA (shRNA) strains to knockdown genes and score the effect on wing growth. This collection of shRNA fly strains was made by the Drosophila RNAi Screening Center (DRSC) and obtained from the Bloomington Drosophila Stock Center (BDSC) (Cook *et al.* 2010; Ni *et al.* 2011). We screened a subset of the latest generation of inducible shRNA strains (TRiP VALIUM 20 vector) because they are more efficient and have fewer off targets than the previous generation of strains that expressed longer dsRNAs (Table S1) (Ni *et al.* 2011; Heigwer *et al.* 2018). We used a *dpp-GAL4* driver to express these *UAS-shRNAs* in a stripe of cells along the anterior-posterior compartment boundary of the larval wing disc beginning during 2^nd^ instar (Figure 1) (Posakony *et al.* 1990; Staehling-Hampton *et al.* 1999; Matsuda and Affolter 2017). These dpp-expressing cells are fated to form most of a central region of the adult wing between longitudinal veins 3 (L3) and 4 (L4), which we scored for reduced area relative to other intervein areas of the wing and total wing size. We also scored the length, spacing (density), and patterning of adult wing hairs in the L3-L4 intervein region compared to other regions of the same wing (Figure 1). The wing hairs are actin protrusions that emanate from each cell and point distally (Guild *et al.* 2005). Screens for disruption of this pattern have identified genes required for planar cell polarity, whereas longer, more widely spaced wing hairs are phenotypes associated with a switch to a polyploid growth program and large cells (Adler *et al.* 2000; Hanson *et al.* 2005; Olofsson and Axelrod 2014).

We crossed 5,260 *UAS-shRNA* strains to *dpp-GAL4 / TM3 Sb Ser*, and scored adult wing of the *shRNA / +* ; *dpp-GAL4 / +* progeny for L3-L4 intervein region size and wing hair morphology, with the *Sb*^-^ siblings lacking *dpp-GAL4* serving as internal controls (Figure 1, Table S1). Among these 5,260 crosses, 507 resulted in lethality before adulthood specifically for the Sb^*+*^ progeny, indicating that knockdown of those genes in the *dpp-GAL4* pattern was not compatible with life (Table 1, Table S1). In 113 crosses, less than 25% of the expected *dpp-GAL4* (Sb+) class survived to adulthood, which we termed semi-lethal (Table 1, Table S1). Given that *dpp-GAL4* expression is not restricted to the wing disc, it is unclear in which tissues knockdown caused lethality. Among the 4,753 shRNA crosses with adult Sb^+^ progeny, 18 had reproducible effects on the central part of the wing (Figure 2B-S, Table1, Table 2, Table S1). All these shRNA strains reduced the area of the L3-L4 intervein region to varying extents relative to sibling controls, which we categorized as mild (class I), moderate (class II), or severe (class III) effects on tissue growth (Figure 2, Table 2). Although each had reproducible and clear effects on tissue mass in the adult wing, the relative severity of these different shRNA phenotypes should be interpreted with caution given that the strength of RNAi knockdown could differ among them. Nonetheless, it is clear that expression of these 18 shRNA strains compromised the growth of wing tissue in the central *dpp-GAL4* expression domain.

**Table 1:** Summary of screen

Total crosses^1^	Lethal^2^	Semi-Lethal^3^	Viable^4^	Mutant wing phenotype
**5,260**	**507**	**113**	**4,640**	**18**

1: See Table S1 for a complete list of strains.

2: The number of crosses in which *Sb^+^*, *dpp-GAL4* offspring died before adulthood whereas *Sb*^-^ siblings without *dpp-GAL4* survived.

3: The number of crosses in which only 25% of expected *Sb^+^*, *dpp-GAL4* offspring survived to adulthood.

4: The number of crosses in which the *Sb^+^*, *dpp-GAL4* offspring survived to adulthood.

**Table 2:** Genes required for growth^1^

Symbol	Name	Growth defect^2^	wing hairs^3^
*stg*	*string*	III	widely spaced, disordered
*Cdc6*	*Cdc6*	I - III	disordered
*eco*	*Establishment of cohesion*	II - III	mild disorder, a few enlarged
*flfl*	*falafel*	II	mild disorder
*lin-52*	*lin-52*	II	widely spaced, longer, disordered
*pita*	*pita*	III	enlarged girth of base
*smog*	*smog*	II	mild disorder
*Rap1*	*Rap1 GTPase*	I	altered planar polarity
*Cisd2*	*CDGSH iron sulfur domain 2*	I - II	slightly longer, hair tufts
β*NACtes6*	*Nascent-associated complex β-subunit-like*, *testis 6*	II - III	widely spaced, longer, hair tufts
*CG3568*	—	I	mild altered planar polarity
*CG4459*	—	I - III	enlarged girth of base, disordered
*CG8132*	—	I - II	mild disorder, hair tufts
*CG9547*	—	II	mild disorder, denser anterior
*CG12171*	—	II - III	mild disorder
*CG34174*	—	II - III	disorder
*CG34177*	—	I - III	Mild disorder
*CG42516*	—	I - II	Mild disorder, hair tufts

1: See Table S1 for FlyBase gene numbers and dsRNA stock numbers.

2: Size of first posterior wing cell between veins L3 and L4. Class I = mild growth defect, Class II = intermediate growth defect; Class III = severe growth defect.

3: Size, shape, and planar cell polarity of wing hairs.

In addition to effects on tissue growth, knockdown of most of these genes affected the organization of the hairs on the surface on the wing (Figure 2, Table 2). This phenotype included disruption of the planar polarity of the hairs, but the orientation of the hairs appeared random, and it remains unclear whether this phenotype is a direct result of a disruption of planar cell polarity, or the indirect result of aberrant tissue growth and morphogenesis (Figure 2, Table 2) (Olofsson and Axelrod 2014). Knockdown of two genes, *lin-52* and *βNactes6*, compromised growth of the L3-L4 intervein region and resulted in longer wing hairs, suggesting that their knockdown may induce a switch to an endoreplication growth program (Figure 2 F, K, T, U Table 2).

Below, we discuss the genes recovered in this screen, their known or predicted function, their orthologs, and their disease associations. We took advantage of the newest online tools that are available through FlyBase, including the Gene to Function (G2F) application that is based on the DRSC Integrative Ortholog Prediction Tool (DIOPT) (Hu *et al.* 2011; Thurmond *et al.* 2019). We also mined information for orthologs and links to human disease using the Alliance of Genome Resources (Alliance) site, which is a new collaborative effort to harmonize data from six model organism knowledgebases and the Gene Ontology (GO) consortium (Howe *et al.* 2018).

### Cell Cycle and Chromosome Segregation Genes

#### string (stg):

Knockdown of *stg* resulted in severe undergrowth of wing tissue and more widely spaced wing hairs, with a disordered wing hair polarity in the L3-L4 intervein region of the adult wing (Figure 2B, T Table 2). While some wing hairs were longer, the average length was not significantly different from controls (Figure 2B, U). The *stg* gene encodes one of two *Drosophila* orthologs of the Cdc25 phosphatase, which dephosphorylates and activates Cdk1 kinase to promote mitotic entry (Edgar and O’farrell 1990). There are three orthologs of Cdc25 in humans whose increased function and expression have been associated with oncogenesis (Table 3) (Sur and Agrawal 2016). Given these known functions, the undergrowth after *stg* knockdown is likely a manifestation of impaired mitotic entry and cell proliferation (Figure 2B, Table 2).

**Table 3:** Known or proposed functions, orthologs, and disease associations

Symbol	Structure – Function^1^	Human ortholog (DIOPT score) ^2^	Disease Associations^3^
*stg*	Phosphatase, activate Cdk1, Mitotic entry	Cdc25 (12)	Cancer
*Cdc6*	Member pre-RC complex; Initiation of DNA replication	Cdc6 (12)	Meier-Gorlin Syndrome
*eco*	Acetyltransferase; establishment of sister chromatid cohesion in S phase	ESCO1,2 (11)	Robert’s Syndrome
*flfl*	Regulatory subunit protein phosphatase 4; kinetochore integrity; chromosome segregation; morphogenesis	PPP4R3A (14)	Cisplatin sensitivity?
*lin-52*	Subunit of Myb-MuvB / dREAM transcription factor complexes; cell cycle, development, *et al.*	LIN52 (13)	Tumor suppression
*pita*	Chromatin insulator protein	ZNF121 (1) FEZF1 (1)	Kallmann Syndrome
*smog*	G protein-coupled receptor; cell migration; cell shape; morphogenesis	GPR158 (5)	Prostate Cancer
*Rap1*	Ras family GTPase; cell polarity, migration shape; developmental signaling ; morphogenesis	RAP1A (14)	Ovarian cancer
			
*Cisd2*	Iron-sulfur & zinc finger domains; Ca+ homeostasis, autophagy	CISD2 (13)	Wolfram Syndrome 2; Hepatocellular carcinoma
*βNACtes6*	Transcription factor; co-translational chaperone; subcellular protein targeting	BTF3, BTF3L4 (1)	
*CG3568*	?	?	
*CG4459*	Organic ion transporter; drug / toxin metabolism; hormonal signaling; neurotransmission.	SLC22A15? (2)	
*CG8132*	Omega Amidase; converts toxic oxoglutaramate to alpha-ketoglutarate	NIT2 (14)	Tumor Suppressor?
*CG9547*	Glutaryl-CoA dehydrogenase; mitochondrial matrix; lysine and tryptophan metabolism	GCDH (15)	Glutaric Acidemia
*CG12171*	Steroid dehydrogenase	HSD17B14 (4)	
*CG34174*	Cdc7 and Cdk2 associated protein; DNA replication; ATR checkpoint signaling; transcription factor	CINP (3)	Cancer
*CG34177*	Von Willebrand factor type C domain: predicted secreted protein;	MSMB (1)	Prostate cancer
			
*CG42516*	TFIIIC complex; pol III transcription	GTF3C1 (3)	

1: Protein domains and known or predicted function in *Drosophila* and / or other organisms.

2: Human ortholog predictions from DIOPT and Gene to Function (G2F), with match score in parentheses from 1 (weakest) to 15 (strongest).

3: Disease Associations curated by FlyBase, Alliance of Genome Resources, and OMIM.

#### Cdc6:

Knockdown of *Cdc6* resulted in a variably expressive phenotype of mild to severe undergrowth of wing tissue with disordered polarity of wing hairs (Figure 2C, Table 2). *Cdc6* is a subunit of the pre-replicative complex, which binds origin DNA and is required for the initiation of DNA replication from yeast to human (Parker *et al.* 2017). Although Cdc6 protein is essential for DNA replication, its knockdown resulted in viable adults with reduced wing tissue, likely because of partial knockdown (Crevel *et al.* 2005) (Figure 2C, Table 2). This result is analogous to the phenotype of mild, hypomorphic alleles of human *CDC6*, which cause a heritable microcephalic primordial dwarfism known as Meier-Gorlin syndrome (Bicknell *et al.* 2011) (Table 3). Thus, similar to humans, partial impairment of *Cdc6* function in *Drosophila* results in undergrowth of tissues during development.

#### establishment of cohesion (eco):

Knockdown of *eco* had an intermediate to severe effect on tissue growth in the wing L3-L4 intervein region (Figure 2D, Table 2). The *eco* gene encodes an acetyltransferase that has a conserved function in eukaryotes to establish sister chromatid cohesion during S phase (Wang and Christman 2001; Rudra and Skibbens 2013). It associates with the replication fork and acetylates the SMC3 subunit of the Cohesin Complex to promote sister chromatid cohesion of newly replicated DNA behind the fork (Ivanov *et al.* 2002; Zhang *et al.* 2008). Its putative human orthologs are the *ESCO1,2* genes (Table 3). Mutations in *ESCO2* cause Robert’s Syndrome, a heritable undergrowth syndrome characterized by reductions in limb size and craniofacial abnormalities, among other pleiotropic phenotypes (Gordillo *et al.* 1993; Vega *et al.* 2005). The undergrowth of the *Drosophila* wing after *eco* knockdown may be the result of increased chromosome instability and reduced cell proliferation (Figure 2D, Table 2).

#### falafel (flfl):

Knockdown of *flfl* resulted in intermediate effects on wing growth (Figure 2E, Table 2). The *flfl* gene encodes the regulatory 3 subunit of protein phosphatase 4 (PP4) (Gingras *et al.* 2005). In *Drosophila*, *flfl* has been shown to target PP4 to the centromere to regulate kinetochore integrity in mitosis, and its loss of function leads to JNK-dependent cell death (Huang and Xue 2015). The orthologous human protein is predicted to be PPP4R3A, which, similar to FLFL protein in flies, physically associates with other PP4 subunits PPP4C and PPP4R2 (Table 3) (Gingras *et al.* 2005). Mutations in *flfl* confer sensitivity to the chemotherapeutic DNA crosslinking agent cisplatin, which is rescued by transgenes expressing human *PPP4R3A* (Gingras *et al.* 2005). The reduced tissue growth after *flfl* knockdown may result from a combination of impaired mitotic chromosome segregation, altered DNA damage response, and cell death (Figure 2E, Table 2).

#### lin-52:

Knockdown of *lin-52* had a mild to moderate effect on growth and resulted in longer, more widely spaced wing hairs (Figure 2F, T, U, Table 2). The Lin-52 protein is a subunit of the modular Myb-MuvB (MMB) and DREAM transcription factor complexes (Beall *et al.* 2002; Korenjak *et al.* 2004; Lewis *et al.* 2004; Guiley *et al.* 2018). From flies to humans, these conserved complexes activate and repress the expression of a large number of genes that have functions in cell cycle, development, and other processes (Georlette *et al.* 2007; Sadasivam and Decaprio 2013). The subunits of the MMB and DREAM include tumor suppressors and oncogenes whose dysregulation cause cancer (Macdonald *et al.* 2017; Musa *et al.* 2017; Iness and Litovchick 2018). The MMB induces the periodic cell cycle expression of genes that are important for M phase and cytokinesis (Georlette *et al.* 2007; Schmit *et al.* 2007; Wen *et al.* 2008; Debruhl *et al.* 2013; Fischer *et al.* 2016). We had found previously that knockdown of the *Myb* subunit of the MMB in the wing impairs expression of mitotic genes and results in a switch to a polyploid growth program, which, similar to *lin-52* knockdown, resulted in longer wing hairs and reduced tissue mass in the adult wing (Rotelli *et al.* 2019). The similar phenotype of *Myb* and *lin-52* knockdown makes sense in the context of recent structure-function studies that indicate that Lin52 is required for the activating Myb subunit to associate with the MuvB core (Andrejka *et al.* 2011; Guiley *et al.* 2018). A cogent hypothesis, therefore, is that *lin-52* knockdown is impairing the ability of the MMB to induce expression of genes required for mitosis and cytokinesis, resulting in a switch to an alternative polyploid growth program.

### Chromatin regulation

#### pita (pita):

Knockdown of *pita* severely impaired wing tissue growth (Figure 2G, Table 2). The *pita* gene encodes a zinc finger protein that is a subunit of a chromatin insulator complex (Maksimenko *et al.* 2015). The two most similar human proteins are the Zn-finger transcription factors ZNF121 (Amino Acid (AA) Identity (I) = 33%, Similarity (S) = 53%) and FEZF1 (AA I = 28%, S = 40%), although the DIOPT score for orthology is low (1/15) (Table 3). Loss of function alleles of *FEZF1* cause Kallmann Syndrome, which is characterized by defects in development of the hypothalamic-pituitary-gonadal (HPG) axis, resulting in the impairment of gonadal development and the sense of smell (hypogonadotropic hypogonadism-22 with anosmia) (Kotan *et al.* 2014; Topaloglu and Kotan 2016) (Table 3). In *Drosophila*, *pita* regulates gene transcription in part through mediating higher-order chromosome structure (Maksimenko *et al.* 2015; Kyrchanova *et al.* 2017). *pita* mutants also have defects in S phase and reduced expression of the replication protein Orc4 (Page *et al.* 2005). The tissue undergrowth after *pita* knockdown may be a result of these cell cycle defects, but, given its role in global chromatin architecture, could also be the result of other pleiotropic effects on gene expression.

### Development and Morphogenesis

#### smog (smog):

Knockdown of *smog* had an intermediate effect on wing growth (Figure 2H, Table 2). *smog* encodes a G protein-coupled receptor that is required for a number of developmental processes (Kerridge *et al.* 2016). During embryogenesis, *smog* is required for embryonic cell migration and shape changes through its regulation of myosin II activity (Kerridge *et al.* 2016; Simões *et al.* 2017). Given these known functions, the reduced tissue mass in the adult wing after *smog* knockdown could be the result of defective cell shape changes and reorganization during wing growth and / or disc eversion and morphogenesis into the adult wing (Figure 2H, Table 2). The most similar human protein is the G protein-coupled receptor 158 (GPR158) (DIOPT 5/15), a broadly-expressed orphan receptor that participates in neurogenesis and has been associated with prostate development and cancer (Orlandi *et al.* 2015; Patel *et al.* 2015; Condomitti *et al.* 2018) (Table 3).

#### Rap1 GTPase (Rap1):

Knockdown of *Rap1* had a mild effect on wing growth (Figure 2I, Table2). *Rap1* is a member of the RAS superfamily of small GTPases, and regulates the actomyosin cytoskeleton for morphogenetic cell migration, apical-basal polarity, cell adhesion, and cell shape changes in a number of tissues (Knox and Brown 2002; Huelsmann *et al.* 2006; Boettner and Van Aelst 2007; Siekhaus *et al.* 2010; Wang *et al.* 2013). Thus, similar to *smog*, *Rap1* knockdown may impair wing growth through altering actomyosin-mediated cell shape changes during growth and / or morphogenesis of the wing disc into the adult wing (Figure 2I, Table 2). However, *Rap1* also regulates the hippo pathway (Chang *et al.* 2018), and is required for receptor tyrosine kinase signaling in the embryo, eye, and wing (Mishra *et al.* 2005; O’keefe *et al.* 2009; Mavromatakis and Tomlinson 2012), suggesting that disruption of these functions may also contribute to the observed wing phenotype after *Rap1* knockdown. The planar cell polarity of the wing hairs was altered, consistent with previous reports that Rap1 has a function in this process (O’keefe *et al.* 2009) (Figure 2I, Table 2). The closest human protein is RAP1A, which evidence suggests also mediates cell shape, polarity, and migration in a variety of tissues, and is involved in ovarian cancer tumorigenesis and metastasis through stimulating cell proliferation, migration, and invasion (Pizon *et al.* 1988; Lu *et al.* 2016) (Table 3).

### Metabolism and Physiology

#### CDGSH iron sulfur domain 2 (Cisd2):

Knockdown of *Cisd2* had a mild to intermediate effect on wing growth (Figure 2J, Table 2). Wing hairs were slightly longer and often grew in tufts of multiple hairs (Figure 2J, Table 2). As its name implies, the protein encoded by the *Cisd2* gene has an iron-sulfur domain, and is 45% identical and 66% similar to human CISD2 protein, which localizes to the endoplasmic reticulum in human cells. Mutations in human *CISD2* cause Wolfram Syndrome 2, a neurological disorder that presents with progressive blindness and deafness, and is associated with gastrointestinal ulcers and diabetes (Table 3) (Amr *et al.* 2007; Mozzillo *et al.* 2014). *CISD2* is frequently deleted in hepatocellular carcinoma (HCC), and haploinsufficiency for *CISD2* in mice disrupts calcium homeostasis, causes fatty liver disease, and promotes HCC (Shen *et al.* 2017; Shen *et al.* 2018). A previous study of fly *Cisd2* uncovered a genetic interaction with overexpressed Palmitoyl Protein Thioesterase (PPT1), a protein involved in protein degradation within the lysosome, and *ceroid-lipofuscinosis*, *neuronal 3* (*CLN3*), whose ortholog is associated with lysosomal storage disease in humans (Jones *et al.* 2014). Jones and colleagues did not, however, find a mutant phenotype associated with *Cisd2* on its own, using either a *Cisd2* dsRNA transgene or animals homozygous for a transposon insertion allele of *Cisd2* (Jones *et al.* 2014). Whether the wing undergrowth phenotype we observed is indeed caused by *Cisd2* depletion will require further experimentation. If so, the wing phenotype is an entry point to further define the molecular mechanisms of Wolfram Syndrome 2 and hepatocellular carcinoma.

### Translation and protein targeting

#### Nascent-associated complex *β*-subunit-Like, testis 6 (*β*NACtes6):

Knockdown of *βNACtes6* had an intermediate to severe effect on wing growth (Figure 2K, Table 2). It also reproducibly resulted in longer wing hairs that often grew in tufts, phenotypes diagnostic of enlarged polyploid cells (Figure 2K, Table 2) (Katzen *et al.* 1998; Adler *et al.* 2000; Hanson *et al.* 2005). βNACtes6 protein is similar to two βNAC paralogs in humans, Basic Transcription Factor 3 (BTF3) (DIOPT 1/15, AA I = 32% S = 47%) and Basic Transcription Factor 3 Like 4 (BTF3L4) (DIOPT 1/15, AA I = 30%, S = 45%) (Table 3). As their name implies, these human proteins were initially defined as general transcription factors that bind the core promoter (Zheng *et al.* 1990). Subsequent studies showed that this eukaryotic family of proteins also regulate translation and are known as β*NACs* (Wiedmann *et al.* 1994). β*NAC* proteins bind αNAC proteins to form the heterodimeric Nascent-Associated Complex (NAC), which associates with the ribosome where it acts as an ATP-dependent chaperone on actively translating proteins (Lauring *et al.* 1995; Deuerling *et al.* 2019). The NAC also regulates the cellular location of the ribosome, inhibiting the targeting of proteins to the ER and promoting targeting to mitochondria (Lauring *et al.* 1995; George *et al.* 1998). An absence of NAC function causes protein mislocalization and can result in cell death (Deuerling *et al.* 2019).

*βNACtes6* is one of six *βNACtes* paralogs in *Drosophila* that are located in the middle of the arm of the X chromosome (five in cytogenetic region 12E and one in 13D). The names of these *βNAC* paralogs include the suffix testis (tes) because they were previously shown to be highly expressed in the *D. melanogaster* male germline during spermatogenesis where they associate with ribosomes (Kogan *et al.* 2017). However, examination of RNA-Seq data from the modENCODE project indicated that there is a pulse of expression of all six of these paralogs in wandering larval 3^rd^ instar imaginal discs, explaining how knockdown of a gene named for its testis expression could impair growth of wing tissue (Graveley *et al.* 2011). All these paralogs are similar to human BTF3 and BTF3L4, but the protein encoded by the *Drosophila bicaudal (bic)* gene is much more similar to these human proteins (BTF3, DIOPT 12/15; AA I = 63%, S = 71%; BTF3L DIOPT 12/15 AA I = 67% S = 75%)(Markesich *et al.* 2000). Maternal Bic protein is localized to the anterior of the embryo where it establishes anterior identity by repressing the translation of the posterior determinant protein Nanos (Markesich *et al.* 2000). *bic* is widely expressed throughout development, suggesting that in most cells *bic* may be the principal *β* subunit of the *Drosophila* NAC, with the *βNACtes* paralogs likely performing more specialized roles in the testis and imaginal discs (Graveley *et al.* 2011). Given the dual function of other βNAC proteins in transcription, however, it may be that the *βNACtes6* wing phenotype is the result of altered transcription. The reduced wing tissue and enlarged bristles after *βNACtes6* knockdown suggests that it is required for normal growth and may influence the choice between cell proliferation and endoreplication growth programs, a possibility that we explore further below.

### Uncharacterized Drosophila genes

A number of the genes that were required for growth have not been extensively characterized in *Drosophila*, and, therefore, are known only by a Computed Gene (CG) number.

#### CG3568:

Knockdown of *CG3568* had a mild effect on wing growth and hair polarity (Figure 2L, Table 2). CG3568 is predicted to encode a 508 amino acid protein with no identifiable protein domains nor orthologs outside of other Dipteran species. In *D. melanogaster*, modENCODE RNA-Seq indicated that *CG3568* is expressed in multiple tissues at multiple stages of *Drosophila* development (Graveley *et al.* 2011). It is perhaps interesting to note that the CG3568 protein, which has identifiable orthologs only in Diptera, begins with the amino acid sequence “MRSFLY.”

#### CG4459:

Knockdown of *CG4459* resulted in a variably expressive mild to severe undergrowth and wing hair polarity phenotypes (Figure 2M, Table 2). *CG4459* encodes a widely expressed protein with a Major Facilitator Superfamily (MFS) domain characteristic of small solute transmembrane transporters in a variety of organisms (Table 3). The CG4459 protein is weakly similar to a large family of human Solute Carrier 22 (SLC22) paralogs in the human genome, the closest being SLC22A1, (AA I = 20%, S = 39%) (Gründemann *et al.* 1994). This family of human transmembrane proteins are organic cation transporters (OCTs) that mediate transport of various pharmaceuticals, toxins, hormones, neurotransmitters, metabolites, and other small molecules, and, therefore, play important roles in human physiology and pharmacology (Table 3) (Lozano *et al.* 2018; Nigam 2018). Further analysis of *CG4459* may reveal new functions for this family of proteins in developing tissues.

#### CG8132:

Knockdown of *CG8132* resulted in severe defects in wing tissue growth, with some hairs growing in tufts (Figure 2N, Table 2). *CG8132* is predicted to encode an omega-amidase that is highly similar to the human protein Nitralase Family Member 2 (Nit2) (DIOPT 14/15), which belongs to a family of enzymes that cleave carbon-nitrogen bonds (Lin *et al.* 2007) (Table 3). Evidence suggests that this omega-amidase removes potentially toxic intermediates by converting alpha-ketoglutaramate and alpha-ketosuccinamate to biologically useful alpha-ketoglutarate and oxaloacetate, respectively, but the *in vivo* functions of this enzyme are controversial (Jaisson *et al.* 2009; Krasnikov *et al.* 2009). Other reports have shown that that Nit2 has an effect on cell proliferation and may be a tumor suppressor (Lin *et al.* 2007; Zheng *et al.* 2015). A recent report showed that knockdown of *CG8132* also strongly impaired growth and development of the *Drosophila* eye (Pletcher *et al.* 2019). Further characterization of the eye and wing phenotypes in flies will further define *CG8132* / *Nit2* cellular functions.

#### CG9547:

Knockdown of *CG9547* had an intermediate effect on wing growth (Figure 2O, Table 2). There was also a reproducible higher density of darkly pigmented wing hairs in the anterior part of the L3-L4 intervein region (Figure 2O, Table 2). The CG9547 protein is highly similar to human Glutaryl-CoA Dehydrogenase (GCDH) (DIOPT 15/15) (Table 3) (Goodman *et al.* 1995). This enzyme is a homotetramer that localizes to the mitochondrial matrix and is involved in lysine and tryptophan metabolic processes (Lenich and Goodman 1986; Goodman *et al.* 1995; Schmiesing *et al.* 2014). In a number of different human populations, alleles of *GCDH* cause the metabolic disorder glutaric acidemia type I, an early-onset neurodegenerative disorder (Table 3) (Goodman *et al.* 1995; Hedlund *et al.* 2006; Schmiesing *et al.* 2017; Schmiesing *et al.* 2018). In *Drosophila*, expression of *CG9547* is upregulated in response to starvation and oxidative stress, and its knockdown altered eye growth (Fujikawa *et al.* 2009; Gruenewald *et al.* 2009; Pletcher *et al.* 2019).

#### CG12171:

Knockdown of *CG12171* had intermediate to severe effects on wing growth (Figure 2P, Table 2). The CG12171 protein is predicted to be a steroid dehydrogenase with similarity to the human steroid dehydrogenase called Hydroxysteroid 17-beta dehydrogenase 14 (HSD17B14) (DIOPT 4/15) (Table 3) (Lukacik *et al.* 2007; Letunic and Bork 2018; El-Gebali *et al.* 2019; Mitchell *et al.* 2019). Evidence suggests that HSD17B14 is involved in steroid catabolic processes and acts on a number of sterols including estradiol, testosterone, fatty acids and prostaglandins (Lukacik *Et Al*., 2007). High throughput protein interaction screens in flies showed that CG12171 protein physically interacts with proteins encoded by *CG31549* and *CG31548* genes, both of which are also predicted to have steroid dehydrogenase activity (Guruharsha *et al.* 2011). The protein products of these three genes are highly similar (69–81% pairwise amino acid identity), and the genes are clustered together at one locus on chromosome 3R, suggesting that they are paralogs that arose through gene duplication and have related functions in steroid biochemistry. Investigating the function of these three genes may reveal novel insights into how cell autonomous regulation of steroid biochemistry mediates tissue growth and differentiation (Figure 2P, Table 2).

#### CG34174:

Knockdown of *CG34174* had intermediate to severe effects on wing growth (Figure 2Q, Table 2). *CG34174* encodes a small protein of 217 AA that is weakly similar to human Cdk2 Interacting Protein (CINP) (AA I = 23% S = 40%) (Table 3). The human CINP protein was initially identified by virtue of binding to the essential S phase kinases Cdc7 and Cdk2 (Grishina and Lattes 2005). That study also provided evidence that CINP is phosphorylated by Cdc7 and physically associates with subunits of the origin recognition complex (ORC) and mini chromosome maintenance (MCM) complex, leading to the hypothesis that CINP has a direct role in DNA replication (Grishina and Lattes 2005). A subsequent study showed that CINP is required for the DNA damage response and G2 cell cycle arrest that is mediated by the ATR-ATRIP checkpoint kinase (Lovejoy *et al.* 2009). That study showed that CINP physically interacts with ATR-ATRIP but did not find evidence for a physical interaction between CINP and CDK2 or Cdc7 (Lovejoy *et al.* 2009). A recent study reported a physical interaction between CINP and the oncogene transcription factor Kruppel-like factor 5 (KLF5), and showed that CINP knockdown suppressed the transcriptional, cell cycle, and tumor promoting effects of KLF5 overexpression, leading the authors to conclude that CINP is a KLF5 transcriptional coactivator (Wu *et al.* 2019). Thus, it is possible that the CINP protein moonlights in multiple cellular processes. Further analysis of *CG34174* will inform which of these function(s) are important for cell proliferation and tissue growth *in vivo*.

#### CG34177:

Knockdown of *CG34177* had severe effects on tissue growth (Figure 2R, Table 2). It is predicted to encode a small protein of 107 AA with a von Willebrand factor C‐domain that is often found in secreted proteins (Sheldon *et al.* 2007). The most similar protein in humans is the secreted protein Microseminoprotein beta (MSMB) (Mbikay *et al.* 1987), but the DIOPT score is low (1/15), with the fly and human proteins being 23% identical and 32% similar (Table 3). However, MSMB protein sequence is known to be rapidly evolving in primates, suggesting that CG34177 may indeed be an ortholog of it. Further, human MSMB protein is expressed in the prostate, while fly CG34177 protein is expressed in the accessory gland, the fly analog of the mammalian prostate, with both proteins being secreted into seminal fluid in flies and mammals (Mbikay *et al.* 1987; Sitnik *et al.* 2016). Lower levels of expression and allelic variants of the *MSMB* gene have been associated with prostate cancer (Harries *et al.* 2010; Waters *et al.* 2010; Lou *et al.* 2012; Peng *et al.* 2017; Bergström *et al.* 2018). *MSMB* and *CG34177* are expressed in tissues other than the prostate and accessory gland, including larval imaginal discs, consistent with its knockdown reducing growth of the wing, but the functions of the human and fly proteins have not been defined (Ulvsbäck *et al.* 1989; Graveley *et al.* 2011).

#### CG42516:

Knockdown of *CG42516* resulted in a mild to intermediate wing undergrowth phenotype, with some hairs growing in tufts (Figure 2S, Table 2). CG42516 protein is weakly similar to human general transcription factor IIIC subunit 6 (GTF3C6) (DIOPT 3/15, AA I = 22%, S = 41%) (Table 3). GTF3C6 is a subunit of the small nuclear RNA (snRNA) activating protein complex, which is required to recruit RNA pol III to promoters of small nuclear RNA genes, including 5S RNAs and tRNAs (Dumay-Odelot *et al.* 2007). A cogent hypothesis, therefore, is that knockdown of *CG42516* impairs growth because of reduced expression of small RNAs that participate in protein translation and other essential cellular processes.

### Knockdown of *βNACtes6* and *lin-52* induces a switch to the endoreplication growth program

One motivation for the screen was to identify genes that influence the decision between mitotic cell proliferation and the polyploid endoreplication growth program. We therefore screened for more widely spaced and longer wing hairs, a phenotype associated with larger polyploid cells (Adler *et al.* 2000; Hanson *et al.* 2005; Olofsson and Axelrod 2014). Knockdown of *stg* resulted in more widely spaced wing hairs, while *lin-52*, *and βNACtes6*, resulted in both more widely spaced and longer wing hairs, suggesting that cells in these wings may have switched to an endoreplication growth program (Figure 2 B, F, K, Table 2). To address whether cells in these and the other 15 gene knockdowns switched to endoreplication, we measured the nuclear size and DNA content of cells in the late third instar larval wing discs. Specifically, we measured the nuclear area and total DAPI fluorescence intensity of wing disc cells in the central *dpp-GAL4* ; *UAS-shRNA* expression domain, identified by co-expression of *UAS-mRFP* (RFP+), and normalized it to the average nuclear area and fluorescent intensity of control, mRFP-negative cells (RFP-) in the wing pouch region of the same wing disc. *UAS-RFP / +*; *dpp-GAL4* / + control animals had nuclei that were of similar size and DNA content in the RFP+ and RFP- cells, and whose average we normalized to 1 (Figure 3A, A’, E). The range of DAPI fluorescent intensity in these control cells ranged from 0.75 to 1.5, likely representing cells in G1 (2C DNA content) and G2 (4C DNA content). Relative to these wild type controls, knockdown of most genes did not significantly increase nuclear size or DNA content in the *dpp-GAL4* expression domain (*P* > 0.01 by *t*-test) (Figure 3E).

Knockdown of *stg* resulted in a central stripe of wing cells with more widely spaced nuclei that appeared less brightly stained with DAPI (Figure 3B, B’). Quantification showed that *stg* knockdown did indeed increase nuclear size (*P* < 0.01), but not total DNA content (measured on both widefield and confocal microscope platforms) (Figure 3E). This result is consistent with previous reports that *stg* mutant wing disc cells arrest at G2 / M and continue to grow in size without replicating their DNA (Neufeld *et al.* 1998). Our results are consistent with the hypothesis that continued cell growth during a G2/M arrest is associated with increasing size of the nucleus without DNA replication, explaining why the total DAPI intensity per nucleus did not increase, but the DAPI brightness / area was lower in these enlarged nuclei. Consistent with this hypothesis, most *stg* knockdown cells had a relative DAPI fluorescence of 1.5, which would correspond to cells in G2 with a 4C DNA content. These results suggest that an increase in nuclear and wing hair size can occur through cell growth without polyploidization.

In contrast, *βNACtes6* or *lin-52* knockdown increased both nuclear size and DNA content (Figure 3C-D’, E). This result suggests that knockdown of these genes induces cells to switch from mitotic proliferation to a polyploid endoreplication program through which tissues grow by an increase in cell size (hypertrophy) rather than cell number, consistent with the observed enlarged wing hair phenotype in adults (Figure 2K, Table 2). It is known that the Lin52 protein is required for the Myb subunit to associate with the core of the MMB transcription factor complex (Andrejka *et al.* 2011; Guiley *et al.* 2015; Guiley *et al.* 2018). This *lin-52* phenotype is, therefore, consistent with our previous finding that knockdown of *Myb* switches wing and other cells to endoreplication (Rotelli *et al.* 2019). Similar to *Myb* knockdown, it is likely that knockdown of *lin-52* impairs the induction of mitotic gene expression by the MMB and promotes a switch to endoreplication cycles that skip mitosis (Rotelli *et al.* 2019). Knockdown of *βNACtes6* or *lin-52* also reduced the area of the L3-L4 intervein region in adult wings, suggesting that tissue growth through an increase in cell size did not fully compensate for growth by cell proliferation (Figure 2F, K, Table 2).

### Conclusion

We have identified 18 *UAS-shRNA* TRiP strains that compromise growth of the wing. Ten of the genes targeted by these *UAS-shRNA* strains have known functions in *Drosophila*, whereas eight genes have not been previously characterized. All but one of these 18 genes are similar to human genes, many of which have been associated with disease. Our results suggest that reduced expression of two genes, *βNACtes6* and *lin-52*, promotes a switch to endoreplication growth program. A switch to endoreplication after *lin-52* knockdown is consistent with our recent finding that repression of a CycA – MMB – AurB pathway promotes endoreplication. *βNACtes6* has a conserved function to regulate translation and protein trafficking, but it is unclear how this is linked to the decision of tissues to grow through an increase in cell size or cell number. While the molecular function of most of the proteins encoded by the 18 genes recovered in this screen have either been described or can be inferred, many have not been fully evaluated for function in developing tissues. Among important questions that remain are how these genes affect cell division, cell death, differentiation, and the accumulation of tissue mass. Further analysis of these genes in *Drosophila* will be a model for defining their function in tissue growth, and how their dysfunction contributes to disease.

The genes identified in this screen fall into a number of broad functional classes, including cell cycle, chromosome segregation, morphogenesis, metabolism, steroid biochemistry, transcription, and translation. Not unexpectedly, five genes whose knockdown affected growth have functions in cell cycle and / or chromosome duplication / segregation (*stg*, *eco*, *flfl*, *cdc6*, *CG34174*). Further study of *CG34174* will help to sort out which of the many functions ascribed to its human ortholog, CINP (DNA replication, damage checkpoint, transcription) are relevant to its function *in vivo* (Grishina and Lattes 2005; Lovejoy *et al.* 2009; Wu *et al.* 2019). Six of the genes fall into the broad class of metabolism and / or organismal physiology (*Cisd2*, *CG12171*, *CG4459*, *CG8132*, *CG9547*, *CG4459*). Notably, a recent candidate shRNA screen of genes with known or predicted metabolic function showed that *CG8132*, *CG9547*, and *CG4459* also influence growth of the *Drosophila* eye disc (Pletcher *et al.* 2019). The cellular activity of these metabolic genes *in vivo* remains incompletely defined, and an important question is whether their activity is similar among all cells or modulated in concert with the development and function of different cell types.

βNACtes6 shRNA expression induced a switch to an endoreplication growth program. The *βNACtes6* shRNA is not predicted to affect the expression of the other *βNAC* paralogs (five *βNACtes* and *bic**)* (Table S1). In addition to their high level of expression during spermatogenesis, all six *βNACtes* genes are expressed in imaginal discs, while two of them are also expressed during late embryogenesis (Roy *et al.* 2010). An important question is whether these different paralogs have tissue specific functions for regulating translation and protein trafficking. Future genetic analysis with loss of function alleles and molecular assays will be required to sort out the division of labor among these paralogs. Our findings lead us to hypothesize that at least *βNACtes6* regulates translation and / or trafficking of a protein that is required for mitotic cell cycles in imaginal discs, and that in the absence of this mitotic function cells switch to alternative endoreplication cycles. Given that the human βNAC orthologs are also transcription factors, it is possible that βNACtes6 influences cell cycle choice by regulating transcription. Investigation of the function of βNAC proteins in *Drosophila* will provide new insights into the function of this family of proteins and their influence on alternative growth programs in development.
